# The distribution of hatching time in *Anopheles gambiae*

**DOI:** 10.1186/1475-2875-5-19

**Published:** 2006-03-22

**Authors:** Alpha S Yaro, Adama Dao, Abdoulaye Adamou, Jacob E Crawford, José MC Ribeiro, Robert Gwadz, Sekou F Traoré, Tovi Lehmann

**Affiliations:** 1 Malaria Research and Training Center, 1805, Point G. Bamako, Mali; 2 Laboratory of Malaria and Vector Research, NIAID, NIH. 12735 Twinbrook Parkway, Rockville, MD, USA

## Abstract

**Background:**

Knowledge of the ecological differences between the molecular forms of *Anopheles gambiae *and their sibling species, *An. arabiensis *might lead to understanding their unique contribution to disease transmission and to better vector control as well as to understanding the evolutionary forces that have separated them.

**Methods:**

The distributions of hatching time of eggs of wild *An. gambiae *and *An. arabiensis *females were compared in different water types. Early and late hatchers of the S molecular form were compared with respect to their total protein content, sex ratio, development success, developmental time and adult body size.

**Results:**

Overall, the distribution of hatching time was strongly skewed to the right, with 89% of the eggs hatching during the second and third day post oviposition, 10% hatching during the next four days and the remaining 1% hatching over the subsequent week. Slight, but significant differences were found between species and between the molecular forms in all water types. Differences in hatching time distribution were also found among water types (in each species and molecular form), suggesting that the eggs change their hatching time in response to chemical factors in the water. Early hatchers were similar to late hatchers except that they developed faster and produced smaller adults than late hatchers.

**Conclusion:**

Differences in hatching time and speed of development among eggs of the same batch may be adaptive if catastrophic events such as larval site desiccation are not rare and the site's quality is unpredictable. The egg is not passive and its hatching time depends on water factors. Differences in hatching time between species and molecular forms were slight, probably reflecting that conditions in their larval sites are rather similar.

## Background

Over 70% of the 500 million malaria cases that occur every year worldwide and even a higher fraction of the mortality burden are concentrated in tropical Africa [1]. Malaria transmission is driven by the mosquito vector system, which in most of sub-Saharan Africa consists of three primary species, namely *Anopheles gambiae*, *Anopheles arabiensis *and *Anopheles funestus*. Both *An. gambiae *and *An. funestus *are further subdivided into semi-isolated populations, typically referred to as forms [2-9]. The extent of genetic isolation between forms was studied extensively [10-13], but the mechanism of isolation and the driving forces are poorly understood. Associations between abundance of certain species and forms in relation to aridity and to rice cultivation have been observed [5,6,14], but the adaptive differences between forms have yet to be identified [15].

The ecology of the egg of African anophelines is a neglected area of study. The conventional view is that hatching occurs as soon as the embryo completes its development [16,17], which takes approximately two days in 27°C when the egg is kept on water. The time from oviposition to hatching of eggs of the molecular forms of *An. gambiae *and their sibling species, *An. arabiensis *was measured in different types of water. This distribution of hatching time was considered as a trait that affects the prospects of larval success had all of them hatched in the same time. If events such as short term desiccation of larval sites are likely, there may be an advantage to delayed hatching of some of the eggs, so they could hatch after the site is filled with water again as is the case in many floodwater species of *Aedes *and *Psorophora*. Similar to these culicines, eggs of the floodwater anopheline, *An. diluvialis *also spread their hatching in distilled water with less than 20% of the eggs hatching during the first 14 d post oviposition (p.o.). [18]. Eggs of *An. gambiae *can tolerate desiccation in humid conditions for several days [19,20]. Flooding of larval site, predator attack on hatching larvae, competition in crowded sites, etc. may also act as selective forces favouring delayed hatching of some of the eggs.

*An. arabiensis *and the molecular forms of *An. gambiae *mostly overlap in their larval sites when sympatric [21,22], but some differences are known. In Mali and Burkina Faso, the M form predominates in permanent sites such as rice fields and much of its range covers dry savannas and the Sahel, where rain is less frequent and predictable than in wet savannas and forest areas, where the S form predominates [6]. *An. arabiensis *is also abundant in drier environments, but it is not common in rice cultivation areas in West Africa. These differences in the type of larval sites, lead us to predict that the hatch distribution varies between the species and the molecular forms.

## Materials and methods

### Mosquito collection

Indoor resting mosquitoes were collected using aspirators during the end of October and early November 2004 from three villages in Mali: Donéguébougou (West 7° 59'5" longitude and North 12° 48'38" latitude), located 15 km north of Bamako, Pimperena (West 5° 42' longitude and North 11° 28' latitude), located 280 km southwest of Bamako, and Selingué (West 8° 17' longitude and North 11° 42' latitude), located 120 km south of Bamako. Mosquitoes were transported to the insectary at the Malaria Research and Training Center in Bamako, which maintains constant 27°C temperature and relative humidity of 75% to 85%. The blood fed and gravid females were placed in one gallon cages and provided with sugar solution. On the third day after collection, each female was placed in a 50 ml Falcon tube containing 15 ml de-ionized water for oviposition. A strip of filter paper (2 cm wide) surrounded the water edge to collect the eggs.

The species and the molecular form of each female (desiccated the morning after oviposition) was determined using PCR assays [2,23]. The eggs were counted on the filter paper, separated into groups of 40–50 eggs (exact count of each dish was recorded) and placed in a labeled Petri dish (50 mm diameter × 9 mm high) containing (i) rice field water, (ii) puddle water (iii) rock pool water, and (iv) de-ionized laboratory water. Egg batches with fewer than 80 eggs were excluded and egg batches with less than 120 or 160 eggs were used for two or three water types, respectively.

### Water collection

Water was collected from natural larval sites that contained early (first and second) and late (third, fourth or pupae) instars of *An. gambiae *s.l. larvae. The rock pool water was collected from Banambani (3 km from Donéguébougou). The depression in the rock (approximately 70 cm diameter and 20 cm deep), was covered with algae, but had no vegetation and the water, filled from a receding stream, was clear. The puddle and rice-field water were collected from Selingué. The puddle, filled with rain water was shrinking in size since the last rain fell 2–3 weeks before the first sampling. It measured approximately 2 m in diameter and 80 cm maximal depth at the first time it was sampled, and during the subsequent five days it shrunk to approximately 1.5 m diameter and 60 cm maximal depth. Its water was cloudy greenish with algae (no vegetation) and had high density of anopheline larvae. The rice field water was collected from shallow canal (approximately 300 m long, 50 cm wide and 25 cm deep) containing slow flowing clear water and surrounded by plants (up to 30 cm high above the water). Underwater vegetation was present as well. A two litre Thermos container was filled with water from each natural larval site every five days and kept in 7°C throughout the experiment. No attempt was made at chemical analysis of the various waters.

### Hatch distribution

The Petri dishes containing the eggs were checked daily. If one or more first stage instar larvae (L1) were observed, the dish was placed over a white background under illumination and photographed using a digital camera (HP Photosmart 945). Pictures were immediately evaluated to ensure that the image was clear and the Petri dish with its label of identification and time was fully included in the photograph. Counting of L1 was done on a computer screen. The number of eggs hatched on each day was calculated as the difference between the current and previous counts of L1. After the 7^th ^day p.o., newly hatched L1 were counted and removed by a Pasteur pipette. Water of the same source was added to the Petri dish every 4–5 days. The date of oviposition was considered to be the day before the egg batch was found.

### Early vs. late comparison

Egg batches of a separate collection from Pimperena were used to compare larvae and adults produced by the eggs that hatched early and late. After the eggs were counted, they were placed in Petri dishes (10 cm diameter × 2 cm high) with de-ionized water and were inspected every two hours, beginning in the second day p.o. for hatching. Newly hatched L1, less than two hours old, were counted and removed. Ten early hatchers (the last of every four L1) were preserved in 85% ethanol, and additional 30 (the first three of every four L1) were placed in a Petri dish (100 mm diameter 20 mm high) filled with 45 ml de-ionized water and raised to adults as described below. Starting when approximately 50 eggs remained to hatch, the same procedure was repeated with the late hatchers. Collection of late hatchers ended when 40 L1 were obtained or after 36 hrs from the time the first L1 of this group was collected. In few cases, less than 30 L1 were obtained for the late hatchers group and the number of early hatchers group in that family was thinned to equal that of the late hatchers. The difference between the midpoint time between the beginning and end of the collection of L1 for each group (early vs. late) was calculated and families whose late hatchers were collected over 8 hrs after collection of the early hatchers were included in subsequent analyses. Larvae were fed daily 0.1 gram of ground Rich Mix of TetraMin Fish Food. Pupae were collected daily and the date of adult emergence was recorded. Adults were preserved in 85% ethanol 24 hr after emergence.

Adult body size was measured by its wing length (the distance from the alular notch to the wing's distal tip) using a dissecting scope fitted with a millimeter ruler at 20×. A single wing was removed, spread over a small drop of water placed on a microscope slide, and covered with cover slip. All wings were measured to the nearest 0.1 mm using the same microscope and settings.

Total protein content of individual L1 was measured using the Micro BCA protein assay (Pierce Biotechnology, Rockford, IL). Individual L1 were dried using a speedvac prior to rotein extraction by subjecting the samples to two freeze-thaw cycles in 115 μl of freshly prepared sodium hydroxide (NaOH, 50 mM) followed by 12 minutes of vortexing at medium speed (setting five out of 10). Following centrifugation for seven minutes at 14,000 rpm, 110 μl of the supernatant were removed and used in determining protein concentration. Two replicate standard curves were prepared in the same solution using Albumin (provided with the Micro BCA kit). One hundred and ten microliters of the aqueous protein supernatant and standard curve samples, including two NaOH blanks, were loaded into individual microplate wells and processed according to the protocol provided with the Micro BCA assay kit. Cubic regression analysis was performed on the logarithm of the protein concentration and absorbance values to estimate the protein concentration of individual larvae based on the standard curve and blanks of each plate. Total larval protein content was calculated based on the volume in the colorimetric reaction and adjusted to the volume in the extraction. Data were analysed in SAS [24].

## Results

### Hatch distribution

**Figure 1:**
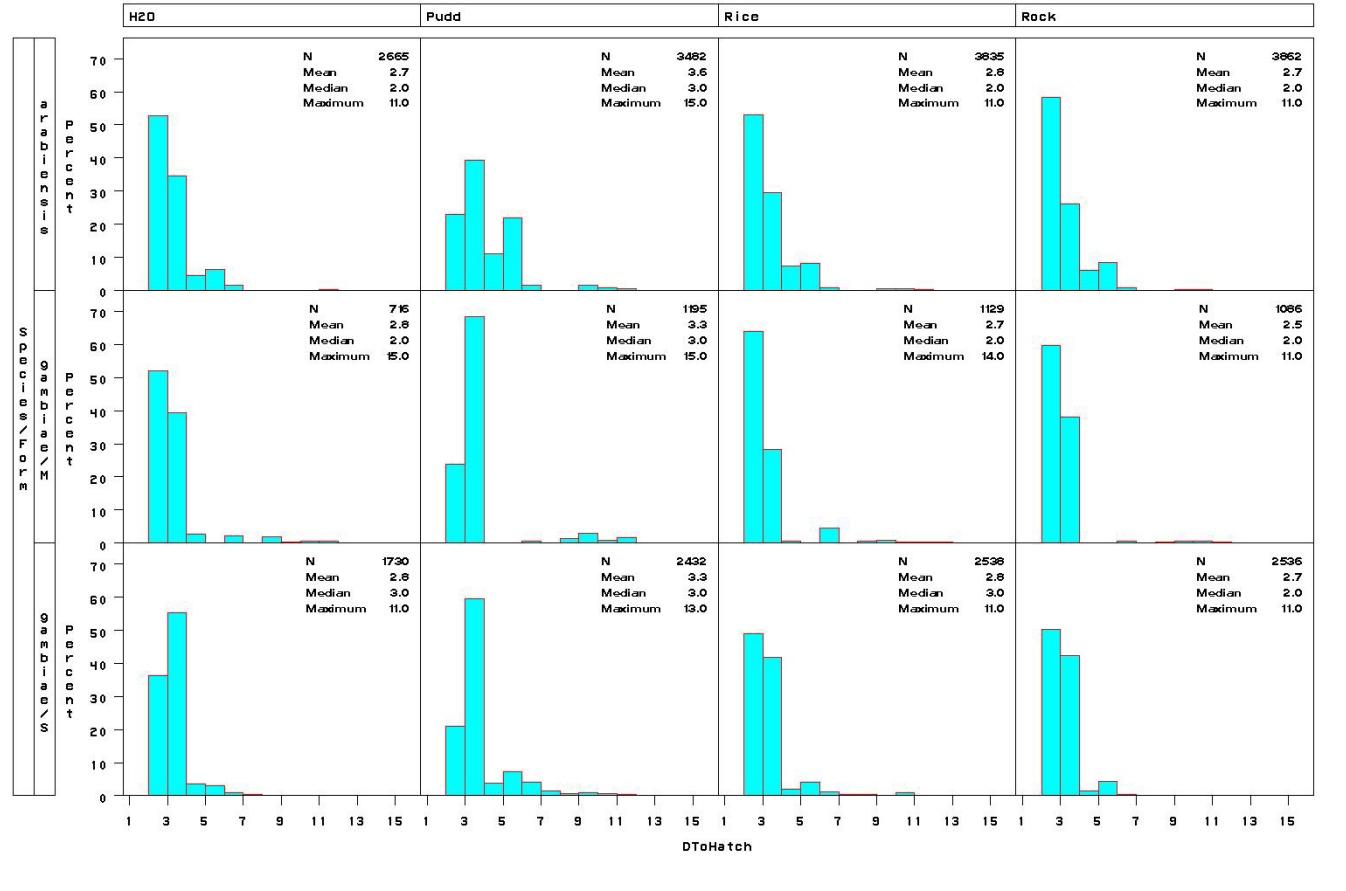
Distribution of egg hatching times in different water types.

Hatching time was measured for a total of 27,206 first instar larvae (L1) comprising 179 egg batches of 85, 32, and 62 of *An. arabiensis*, the M, and the S molecular forms of *An. gambiae*, respectively. In each location one species or molecular form predominated. Thus, 98% of the *A. arabiensis *females were collected in Doneguebougou, 92% of the M form were collected in Selingue, whereas 86% of the S form were collected in Pimperena. Overall hatching rate was 86%. The majority of eggs hatched on the second and the third days p.o. regardless of species, form and water types (Figure 1 and Table 1). However, hatching continued up to two weeks p.o., forming a distribution that is strongly skewed to the right (Figure 1). Late hatchers occurred in most egg batches (84% of the egg batches had one or more eggs hatching at day five p.o. or later) rather than representing few exceptional egg batches. Overall, 89% of the eggs hatched during the first two days (second and third p.o.), 10% hatched during the next 4 days, and the remaining 1% hatched over the subsequent week (Figure 1 and Table 1).

**Table 1:** Hatching time of the eggs of *An. arabiensis *(A) and the molecular forms of *An. gambiae *(M and S) in different water types. Differences between forms in the same type of water are shown (see text for details).

Water		Species	Hatch time (d) p.o.			2 df χ2 tests (**= P<0.01 ***=P<0.001)		
type	N	Form	2–3	4–7	8–15	M – S	M – A	S – A
dH2O	2,665	A	87.3	12.4	0.4	37.7***	85.2***	23.6***
	716	M	91.3	5.0	3.6			
	1,730	S	91.6	7.8	0.6			
Rice	3,835	A	82.6	16.4	1.1	12.1**	100.9***	103.8***
	1,129	M	92.2	5.2	2.6			
	2,538	S	90.1	7.6	1.5			
Rock-pool	3,862	A	84.5	15.0	0.5	70.6***	186.9***	107.6***
	1,086	M	97.8	0.5	1.7			
	2,536	S	92.8	6.6	0.6			
Puddle	3,482	A	62.2	34.7	3.1	237.4***	548.4***	236.8***
	1,195	M	92.2	0.6	7.2			
	2,432	S	80.6	16.8	2.6			

Total	27,206	overall	84.5	13.8	1.7			
Total w/o Puddle	20,097	overall	88.4	10.6	1.0			

To assess the differences in the hatching time between species and molecular forms at different water types, the hatch distribution was divided into three intervals (as described above, Table 1) and subjected to Chi square heterogeneity tests separately in each type of water. Because all four global tests were highly significant (χ^2^>112, df = 4, P < 0.002), pair-wise tests were subsequently conducted and found to be significant as well (Table 1). In all water types, the molecular forms of *An. gambiae *appear more similar to each other compared with *An. arabiensis *in having a higher hatching rate during the early peak of hatching (day 2 and day 3 p.o.) than that of *An. arabiensis*. The main difference between the molecular forms was the higher hatching rate of the S form during the middle period of hatching (day 4 to day 7), whereas the M form had higher hatching rate during the late period (day 8 to day 15). These differences between species and molecular forms were consistent in all water types (Table 1).

The difference between water types was tested in each species separately using contingency table heterogeneity tests (water type × hatching time interval) and found to be highly significant in all cases (χ^2^>139, df = 6, P < 0.001, Figure 1 and frequencies are given in Table 1). The hatching distribution was most distinct in puddle water (Figure 1 and Table 1). Because we used only one source of each type of water, these results provide no information on variation between water from the same type, e.g., different puddles or different rice fields, which may be as large, or larger than that measured here between the different water types. Nonetheless, these results suggest that eggs of these anopheline species change their hatching time according to water type.

### Comparison of early and late hatchers

Early and late hatchers in 26 families of the S molecular form were compared with respect to their total protein content (measured in up to two-hour old L1 that hatched in laboratory deionized water), sex composition, developmental success, developmental time (from hatching to adult emergence) and the size of adults they produce (measured as wing length). Total protein content of early hatchers (0.466 μg/L1) was similar to that of late hatchers (0.472 μg/L1), and the difference was not significant (Wilcoxon sign paired test on family means, n = 19, S = 36, P > 0.15). Females represented 55% of the total adults, but there was no difference in sex ratio between early and late hatchers (χ^2^<1.9, df = 1, P > 0.15, not shown). Development success of early hatchers (78%) was similar to that of late hatchers (83%) and the difference was not significant (Wilcoxon sign paired test by family, n = 26, S = 31.5, P > 0.4). Developmental time of early hatchers was shorter than that of late hatchers (mean values: 10.3 d vs. 11.6 d, respectively, Wilcoxon sign paired test by family, n = 26, S = 175.5, P < 0.002). Finally, body size measured by wing length was smaller in early hatchers than in late hatchers for females (mean values of 2.96 mm vs. 3.10, respectively, Wilcoxon sign paired test by family, n = 21, S = 87, P < 0.001) and males (mean values of 2.82 mm vs. 2.92 mm, respectively, Wilcoxon sign paired test by family, n = 21, S = 71, P < 0.01). These results suggest that the egg batch is not homogenous with respect to the attributes of the individual across different life stages.

## Discussion

Although it lasts only a few days, the egg is an important part of the ecology of African anophelines that has been scarcely studied. The results reveal a complex pattern of hatching of eggs of field collected females and within-batch heterogeneity in egg attributes that are expressed in the egg, larva, and adult stages. Contrary to the convention that the eggs of these anophelines hatch within two days, it was found that hatching spread over more than a week in many egg batches in all water types. In both species, 85–90% of the eggs hatched during the second and third days p.o., 10–14% hatched during the next 4 days, and the remaining 1–2% hatched over the following week. Whether this strategy is adaptive has yet to be determined, but the delayed hatching is potentially protective against the loss of the total batch had all eggs hatched together and experienced larval site desiccation or pathogen/predator attack. On the other hand, longer time to maturity exposes the aquatic stages to increased risk and delays reproduction, which carries clear disadvantages. In small larval sites, competition between larvae may favour staggered hatching, but the delay of mere 10% of the egg batch is unlikely to alleviate it greatly. Thus, the hatch distribution may represent an optimal strategy to balance the "desirable" rapid development into reproductive adults with the likelihood of various events leading to decreased larval success. Spread out hatching over time is clearly beneficial if all eggs are deposited in one or few sites, but it could remain beneficial even if females spread their egg batch over several larval sites if the likelihood of catastrophic events over short period is high. Studies to determine how females distribute their eggs over larval sites and the likelihood of catastrophic events such as larval site desiccation would be helpful to interpret this pattern. Instalment hatching, i.e., the spread of hatching over a series of inundations has long been recognized as a putative adaptation to larval site desiccation in floodwater species of *Aedes *and *Psorophora *with weeks or months between inundations [25-28]). Similar to these culicines, eggs of *An. diluvialis *(previously named *Anopheles quadrimaculatus *C1) that cohabits their larval sites, spread their hatching in distilled water with less than 20% of the eggs hatching during the first 14 d p.o., unless being subjected to a specific hatching stimulus such as vacuum[18]. Thus, delayed hatching in anophelines may be more common than previously believed.

Although sympatric molecular forms of *An. gambiae *and *An. arabiensis *cohabit many larval sites [21,22,29] where their eggs have probably evolved under similar selective pressures, there are habitats that are exploited by one and not by the other form or species. For example, *An. arabiensis *(in East Africa) and the M form of *An. gambiae *commonly use rice fields, whereas the S form is practically absent from rice fields but commonly found in temporary rain filled puddles [6]. On a larger geographical scale, *An. arabiensis *and the M form inhabit dryer areas than those inhabited by the S molecular form. The differences in the hatch distribution between the molecular forms of *An. gambiae *and between them and that of *An. arabiensis *may represent adaptive responses to different expectations of catastrophic events they tend to experience overall. Larger fractions of the eggs of *An. arabiensis *hatched late, consistent with its presence in dry habitats with higher likelihood of larval site desiccation. Knowledge of the occurrence of various catastrophic events (such as larval site desiccation) in different habitats can help evaluate the adaptive value of the observed hatch distribution. Whether different source of blood affect progeny traits is questionable. However in this region, indoor collections of *An. arabiensis *and the molecular forms of An. gambiae exhibited high (>90%) human biting rate [30,31], thus it could not contribute to the variation reported here.

The effect different water types had on hatching time was especially pronounced with the puddle water. Further study is needed to determine whether the egg responds to certain signals that may indicate the likelihood of a catastrophe or to a stressful environment, which prolongs embryonic development. The water was collected from a puddle that had a high density of *An. gambiae *s.l. (and other culicids) larvae in it because it concentrated the larvae from considerably larger area in the course of drying out. Additionally, it was one of the few remaining puddles in the beginning of the dry season. It is possible that the delayed hatching in this water exhibited by all species and forms, was due to a signal the eggs could pick up from this water that signified a rapidly drying larval site. Eggs of many culicine species hatch in response to the reduced concentration of oxygen in the water [16]. Eggs of *An. diluvialis *hatch poorly in distilled water, but they hatch readily in swamp water and in response to unidentified organic chemicals in an extract from swamp soil [32].

Heterogeneity among eggs of the same batch was suggested by differences between early and late hatchers of the S molecular form. Eggs of *Ae. taeniorhynchus *and *Ae. cantator *showed heterogeneity among eggs in the same egg batch, with some of the eggs requiring exposure to cold temperature before hatching, and so delay their hatching to the next summer [27,33]. Although there was no difference between the early and late hatchers of *An. gambiae *in their total protein content, as was their developmental success; late hatchers developed slower into adults and produced larger adults than early hatchers as was found for *Ae. aegypti *[34]. These differences suggest that the egg batch consists of individual larvae that are destined to develop in different ways to maximize various combinations of rapid development and adult size. This mixed strategy may be a response to the unpredictability of the conditions in the larval site. Accordingly, in larval sites with good nutrition and low risk of desiccation or predation, larvae that produce larger adults will have higher fitness despite their longer developmental time, but in sites of poor nutrition and high risk of predation those that develop faster will survive better and have higher fitness despite producing smaller adults. It cannot entirely be ruled out that the late hatchers developed into larger adults because of their density during the first 36 hr of collection was lower due to our collection procedure (see Materials and methods). It is assumed that density effect at this early stage is unimportant. The association between hatching time and development time may reflect a developmental constraint, such as slow vs. fast growth and differentiation. Alternatively, it facilitates the shortest possible developmental time of the aquatic stage. Differences between early and late hatchers were described in a colony of *Ae. aegypti *[35], but the development time was shorter and sex ratio was biased towards females in late hatchers.

## Conclusion

These results reveal that egg batches of *An. gambiae *and *An. arabiensis *consist of heterogenic individuals with respect to traits such as hatching time and larval development time. Slight but significant differences were found between the hatching time distributions of the molecular forms of *An. gambiae *and between them and that of *An. arabiensis*. This similarity probably reflects that the larvae of both species and molecular forms are exposed to similar risks in their larval sites. The "programmed" hatching time can be changed by factors in the water. Further, the eggs in a single egg batch appear to follow different developmental plans. The variation in hatching time and in the larval development within an egg batch may help cope with the unpredictability of conditions in the larval site. Additional studies are needed to better characterize these egg traits, their evolutionary significance, physiological, and genetic basis as well as the stability in key conditions of larval sites.

## Authors' contributions

ASY, AD, AA, and SFT carried out the field and laboratory experiments on the egg hatching and comparison of early and late hatchers. JMCR suggested and helped optimize the protein assay, which was performed by JEC. TL conceived the study and performed the statistical analysis. The experimental design was shaped by TL, ASY, SFT and RG. ASY and TL have written the ms with input from JMCR, JEC, and RG. All authors read the final version and approved it.
